# Complete Chromosome and Plasmid Sequences of Two Plant Pathogens, Dickeya solani Strains D s0432-1 and PPO 9019

**DOI:** 10.1128/genomeA.00233-18

**Published:** 2018-04-26

**Authors:** Slimane Khayi, Pauline Blin, Teik Min Chong, Kok-Gan Chan, Denis Faure

**Affiliations:** a Institute for Integrative Biology of the Cell (I2BC), CEA CNRS Université Paris-Sud, Université Paris-Saclay, Gif-sur-Yvette, France; b Biotechnology Research Unit, National Institute for Agronomic Research (INRA), Rabat, Morocco; c Division of Genetics and Molecular Biology, Institute of Biological Sciences, Faculty of Science, University of Malaya, Kuala Lumpur, Malaysia; d International Genome Centre, Jiangsu University, Zhenjiang, China; e ISB, Faculty of Science, University of Malaya, Kuala Lumpur, Malaysia

## Abstract

Dickeya solani species are emerging bacterial pathogens of Solanum tuberosum. Here, we announce the complete genome sequences of two strains, Dickeya solani D s0432-1 and PPO 9019. Strain PPO 9019 represents the first described member of the genus Dickeya with an extrachromosomal genetic element.

## GENOME ANNOUNCEMENT

The genera Dickeya and Pectobacterium encompass several bacterial species causing blackleg and soft-rot diseases on economically important crops and ornamental plants around the world (1). In Europe, Pectobacterium spp. and Dickeya dianthicola are the most prevalent pathogens associated with the soft-rot and blackleg symptoms of potato cultures. However, since the 2000s, a novel species named Dickeya solani has emerged. Its emergence was associated with an increased incidence of soft-rot and blackleg diseases in several European countries and in the Middle East (2).

The whole genomes of D. solani strains D s0432-1 and PPO 9019 were subjected to Pacific Bioscience (PacBio) sequencing technology to highlight their complete nucleotide sequences. Draft genome sequences of these two strains, obtained using other DNA sequencing technologies, were published previously (3, 4). The genomic DNA was extracted using the MasterPure DNA purification kit (Epicentre, Inc., USA) following the manufacturer's recommendations. Quantification and quality control of the DNA were completed using a NanoDrop device (ND 1000), a Qubit 2.0 fluorometer, and agarose gel electrophoresis at 1.0% (wt/vol). The library insert size was 10 kbp. Before the assembly step, short reads that were less than 500 bp were filtered, and the minimum polymerase read quality used for mapping the subreads from a single zero-mode waveguide was set to 0.75. Totals of 340,825 filtered reads (*N*_50_, 4,976 bp) for D. solani PPO 9019 and 287,091 filtered reads (*N*_50_, 4,319 bp) for D. solani D s0432-1 were used for genome assembly.

The assembly was sequenced using RS_HGAP_Assembly software v2.0. The cutoff lengths of seed bases were set at 10,117 bp for strain PPO 9019 and 8,999 bp for strain D s0432-1 in order to serve as a reference for the recruitment of shorter reads for preassembly. The resulting consensus accuracy based on multiple sequence alignment of the subreads was at 99.99%. Polished assembly resulted in one contig for D. solani D s0432-1 with a length of 4,919,812 bp and 160×  coverage. Two contigs were obtained from the assembly for the D. solani strain PPO 9019, measuring 4,918,850 bp for the chromosome and 43,580 bp for the plasmid, with an average coverage of 220×.

The genome sequences for both strains were submitted for annotation to the NCBI Prokaryotic Genome Annotation Pipeline (released 2013).

### Accession number(s).

The complete genome sequences for these bacteria have been deposited at DDBJ/EMBL/GenBank under the accession numbers CP017454 and CP017455 (Dickeya solani strain PPO 9019) and CP017453 (Dickeya solani strain D s0432-1).
